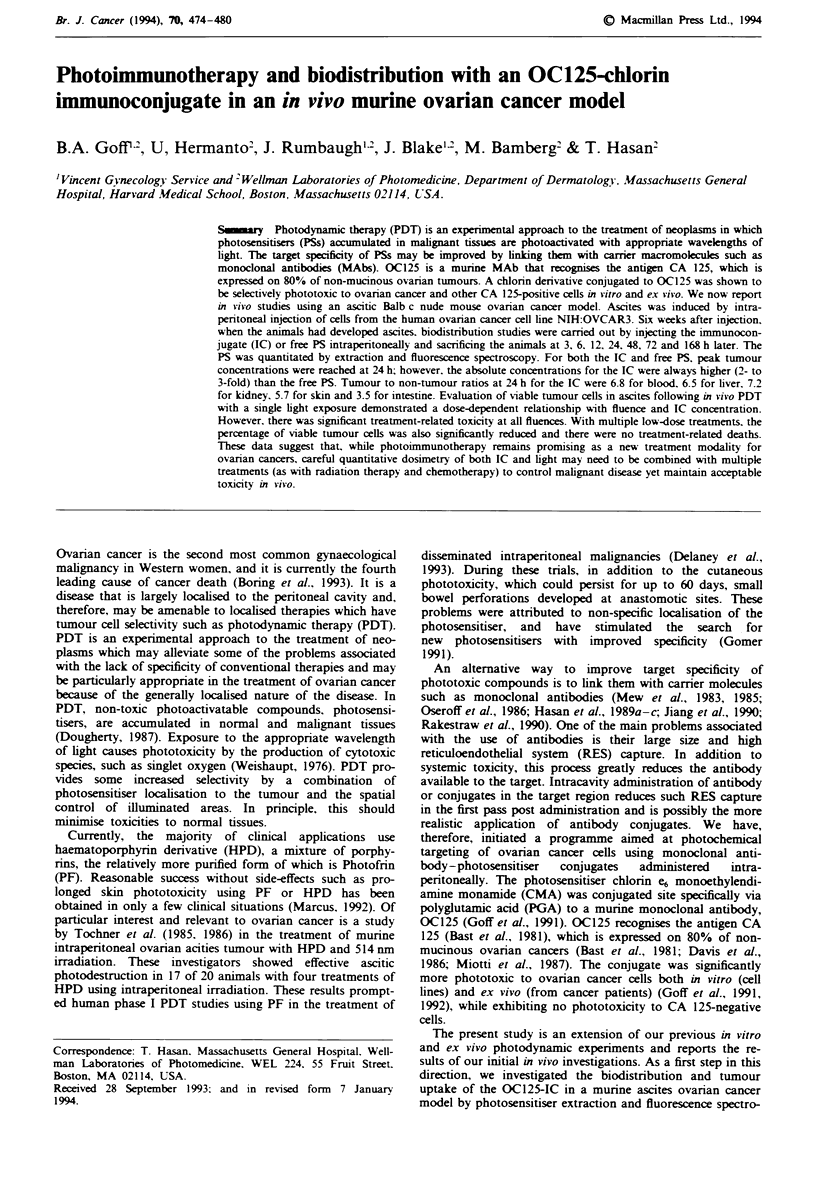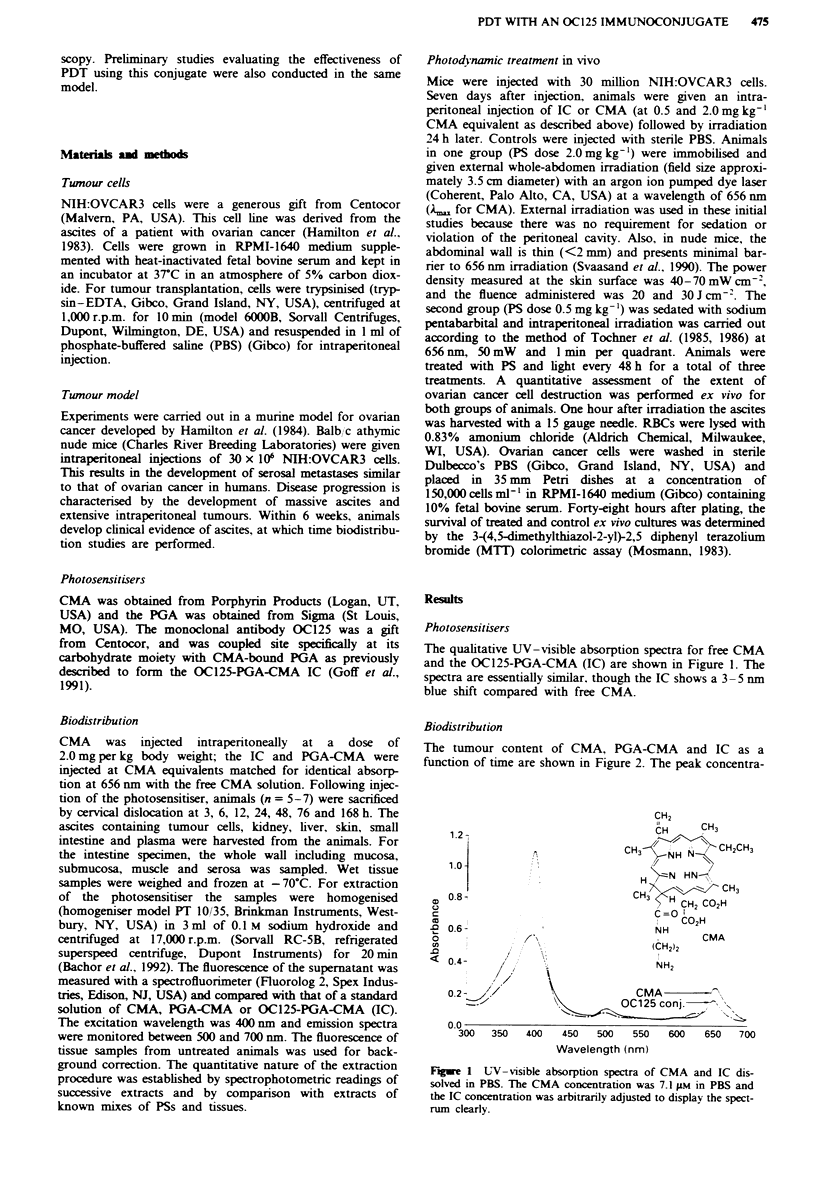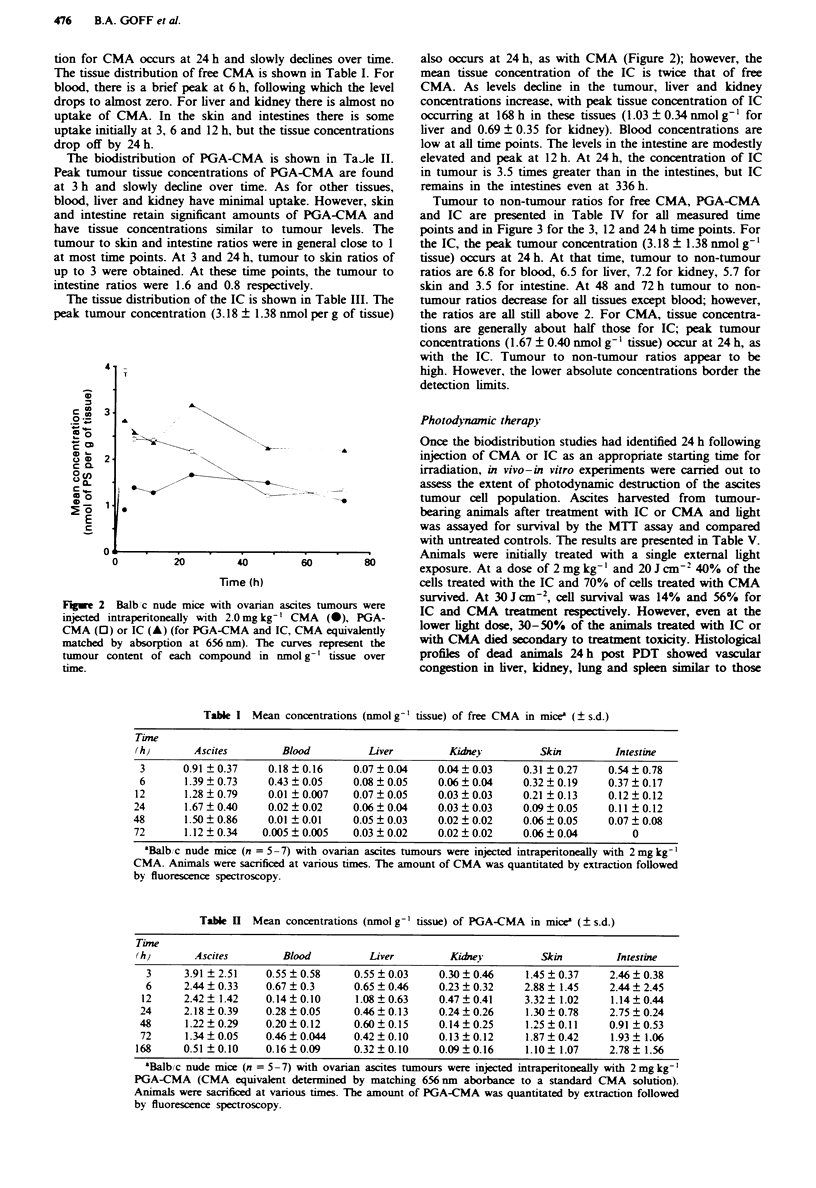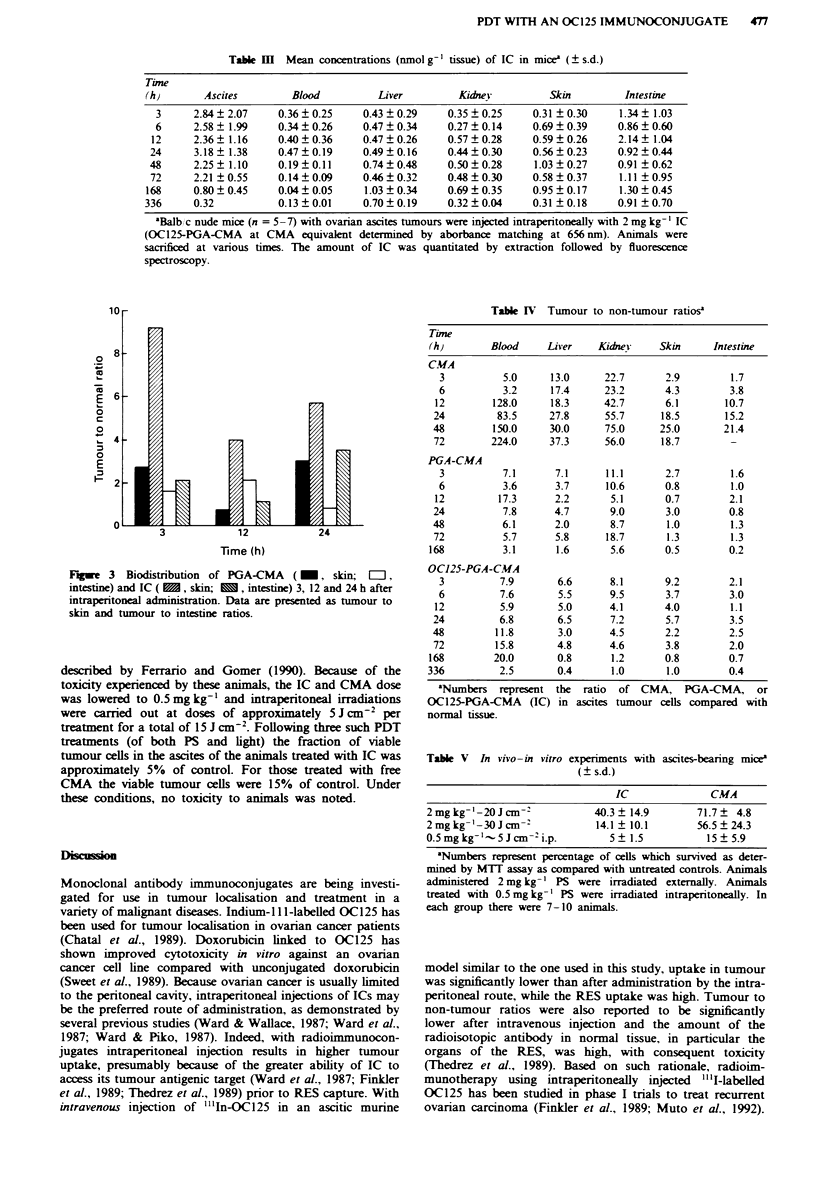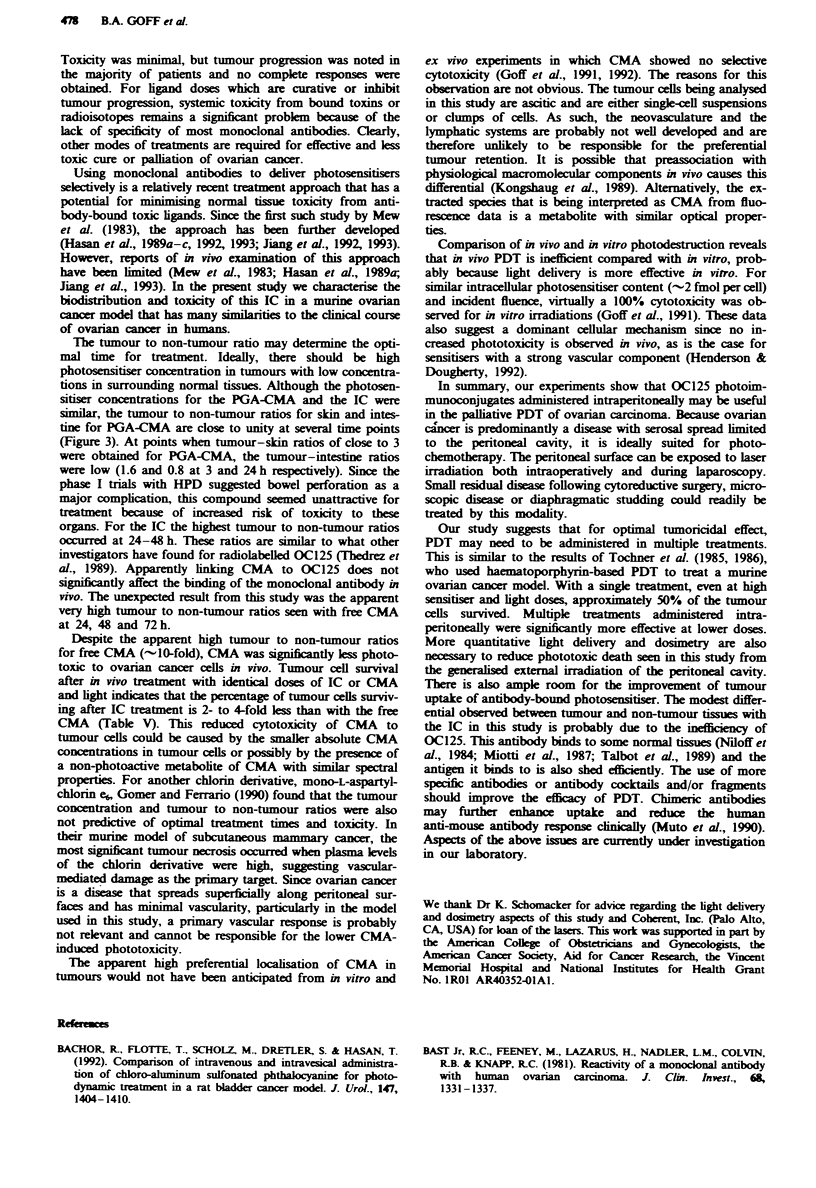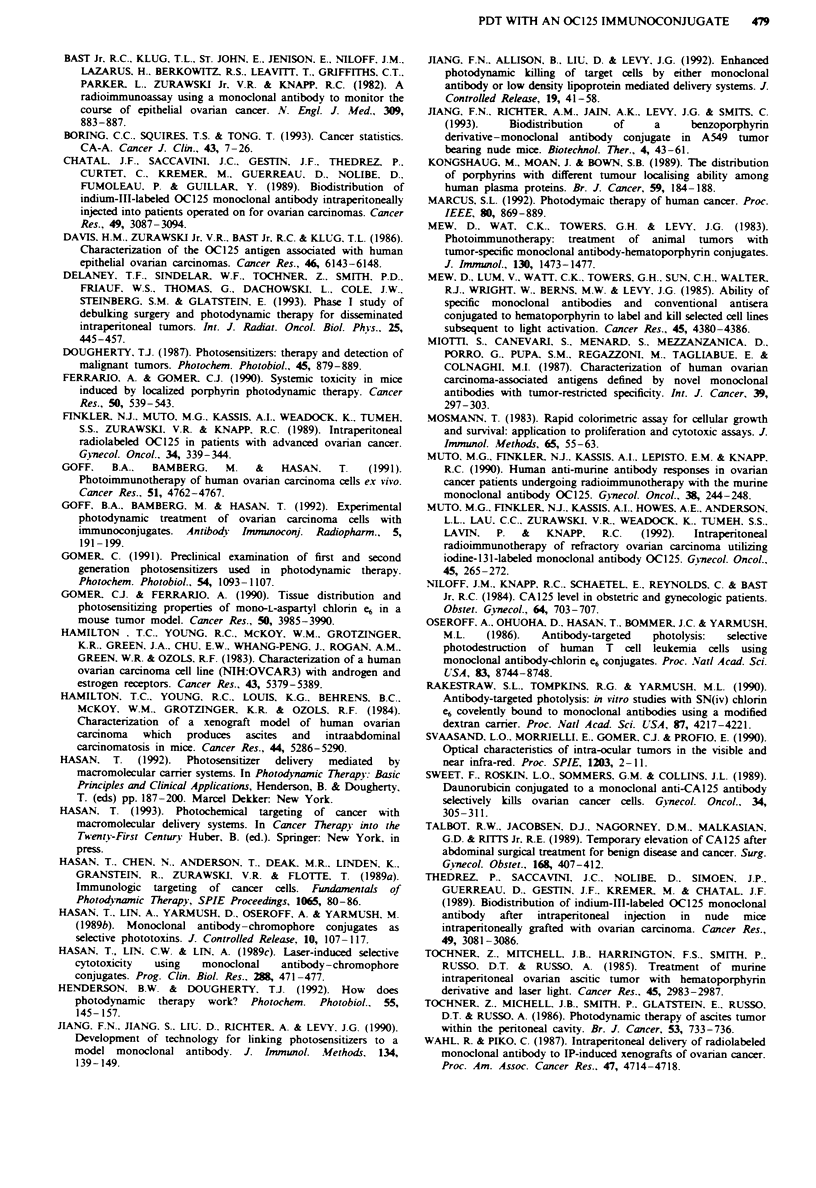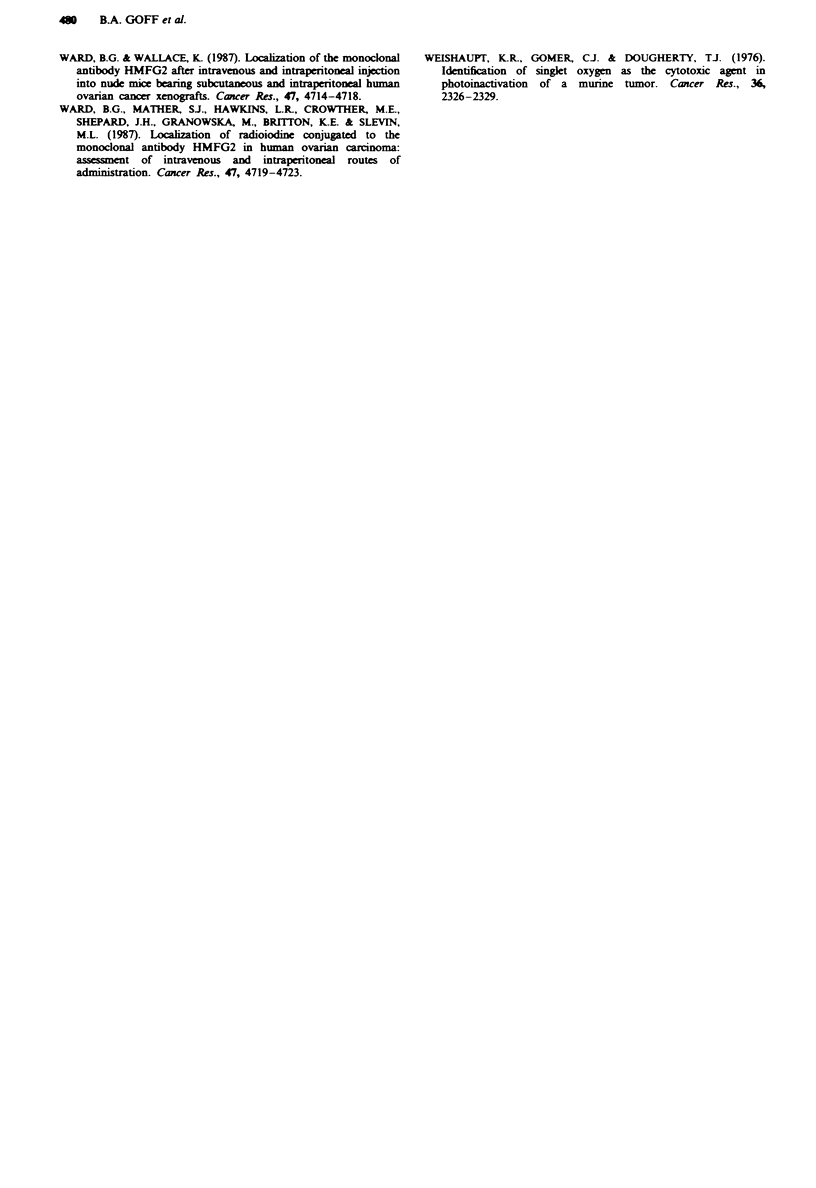# Photoimmunotherapy and biodistribution with an OC125-chlorin immunoconjugate in an in vivo murine ovarian cancer model.

**DOI:** 10.1038/bjc.1994.330

**Published:** 1994-09

**Authors:** B. A. Goff, U. Hermanto, J. Rumbaugh, J. Blake, M. Bamberg, T. Hasan

**Affiliations:** Vincent Gynecology Service, Massachusetts General Hospital, Harvard Medical School, Boston 02114.

## Abstract

Photodynamic therapy (PDT) is an experimental approach to the treatment of neoplasms in which photosensitisers (PSs) accumulated in malignant tissues are photoactivated with appropriate wavelengths of light. The target specificity of PSs may be improved by linking them with carrier macromolecules such as monoclonal antibodies (MAbs). OC125 is a murine MAb that recognises the antigen CA 125, which is expressed on 80% of non-mucinous ovarian tumours. A chlorin derivative conjugated to OC125 was shown to be selectively phototoxic to ovarian cancer and other CA 125-positive cells in vitro and ex vivo. We now report in vivo studies using an ascitic Balb/c nude mouse ovarian cancer model. Ascites was induced by intraperitoneal injection of cells from the human ovarian cancer cell line NIH:OVCAR3. Six weeks after injection, when the animals had developed ascites, biodistribution studies were carried out by injecting the immunoconjugate (IC) or free PS intraperitoneally and sacrificing the animals at 3, 6, 12, 24, 48, 72 and 168 h later. The PS was quantitated by extraction and fluorescence spectroscopy. For both the IC and free PS, peak tumour concentrations were reached at 24 h; however, the absolute concentrations for the IC were always higher (2- to 3-fold) than the free PS. Tumour to non-tumour ratios at 24 h for the IC were 6.8 for blood, 6.5 for liver, 7.2 for kidney, 5.7 for skin and 3.5 for intestine. Evaluation of viable tumour cells in ascites following in vivo PDT with a single light exposure demonstrated a dose-dependent relationship with fluence and IC concentration. However, there was significant treatment-related toxicity at all fluences. With multiple low-dose treatments, the percentage of viable tumour cells was also significantly reduced and there were no treatment-related deaths. These data suggest that, while photoimmunotherapy remains promising as a new treatment modality for ovarian cancers, careful quantitative dosimetry of both IC and light may need to be combined with multiple treatments (as with radiation therapy and chemotherapy) to control malignant disease yet maintain acceptable toxicity in vivo.


Br. J. Cancer (1994), 70, 474-480 © Macmillan Press Ltd., 1994

### Photoimmunotherapy and biodistribution with an OC125-chlorin immunoconjugate in an *in vivo* murine ovarian cancer model

B.A. Goff[1,2], U, Hermanto[2], J. Rumbaugh[1,2], J. Blake[1,2], M. Bamberg[2] & T. Hasan[2]

[1]*Vincent Gynecology Service and* [2]*Wellman Laboratories of Photomedicine, Department of Dermatology, Massachusetts General Hospital, Harvard Medical School, Boston, Massachusetts 02114, USA.*

**Summary** Photodynamic therapy (PDT) is an experimental approach to the treatment of neoplasms in which photosensitisers (PSs) accumulated in malignant tissues are photoactivated with appropriate wavelengths of light. The target specificity of PSs may be improved by linking them with carrier macromolecules such as monoclonal antibodies (MAbs). OC125 is a murine MAb that recognises the antigen CA 125, which is expressed on 80% of non-mucinous ovarian tumours. A chlorin derivative conjugated to OC125 was shown to be selectively phototoxic to ovarian cancer and other CA 125-positive cells *in vitro* and *ex vivo*. We now report *in vivo* studies using an ascitic Balb c nude mouse ovarian cancer model. Ascites was induced by intraperitoneal injection of cells from the human ovarian cancer cell line NIH:OVCAR3. Six weeks after injection, when the animals had developed ascites, biodistribution studies were carried out by injecting the immunoconjugate (IC) or free PS intraperitoneally and sacrificing the animals at 3, 6, 12, 24, 48, 72 and 168 h later. The PS was quantitated by extraction and fluorescence spectroscopy. For both the IC and free PS, peak tumour concentrations were reached at 24 h; however, the absolute concentrations for the IC were always higher (2- to 3-fold) than the free PS. Tumour to non-tumour ratios at 24 h for the IC were 6.8 for blood, 6.5 for liver, 7.2 for kidney, 5.7 for skin and 3.5 for intestine. Evaluation of viable tumour cells in ascites following *in vivo* PDT with a single light exposure demonstrated a dose-dependent relationship with fluence and IC concentration. However, there was significant treatment-related toxicity at all fluences. With multiple low-dose treatments, the percentage of viable tumour cells was also significantly reduced and there were no treatment-related deaths. These data suggest that, while photoimmunotherapy remains promising as a new treatment modality for ovarian cancers, careful quantitative dosimetry of both IC and light may need to be combined with multiple treatments (as with radiation therapy and chemotherapy) to control malignant disease yet maintain acceptable toxicity *in vivo*.

Ovarian cancer is the second most common gynaecological malignancy in Western women, and it is currently the fourth leading cause of cancer death (Boring *et al.*, 1993). It is a disease that is largely localised to the peritoneal cavity and, therefore, may be amenable to localised therapies which have tumour cell selectivity such as photodynamic therapy (PDT). PDT is an experimental approach to the treatment of neoplasms which may alleviate some of the problems associated with the lack of specificity of conventional therapies and may be particularly appropriate in the treatment of ovarian cancer because of the generally localised nature of the disease. In PDT, non-toxic photoactivatable compounds, photosensitisers, are accumulated in normal and malignant tissues (Dougherty, 1987). Exposure to the appropriate wavelength of light causes phototoxicity by the production of cytotoxic species, such as singlet oxygen (Weishaupt, 1976). PDT provides some increased selectivity by a combination of photosensitiser localisation to the tumour and the spatial control of illuminated areas. In principle, this should minimise toxicities to normal tissues.

Currently, the majority of clinical applications use haematoporphyrin derivative (HPD), a mixture of porphyrins, the relatively more purified form of which is Photofrin (PF). Reasonable success without side-effects such as prolonged skin phototoxicity using PF or HPD has been obtained in only a few clinical situations (Marcus, 1992). Of particular interest and relevant to ovarian cancer is a study by Tochner *et al.* (1985, 1986) in the treatment of murine intraperitoneal ovarian acities tumour with HPD and 514 nm irradiation. These investigators showed effective ascitic photodestruction in 17 of 20 animals with four treatments of HPD using intraperitoneal irradiation. These results prompted human phase I PDT studies using PF in the treatment of disseminated intraperitoneal malignancies (Delaney *et al.*, 1993). During these trials, in addition to the cutaneous phototoxicity, which could persist for up to 60 days, small bowel perforations developed at anastomotic sites. These problems were attributed to non-specific localisation of the photosensitiser, and have stimulated the search for new photosensitisers with improved specificity (Gomer 1991).

An alternative way to improve target specificity of phototoxic compounds is to link them with carrier molecules such as monoclonal antibodies (Mew *et al.*, 1983, 1985; Oseroff *et al.*, 1986; Hasan *et al.*, 1989*a*–*c*; Jiang *et al.*, 1990; Rakestraw *et al.*, 1990). One of the main problems associated with the use of antibodies is their large size and high reticuloendothelial system (RES) capture. In addition to systemic toxicity, this process greatly reduces the antibody available to the target. Intracavity administration of antibody or conjugates in the target region reduces such RES capture in the first pass post administration and is possibly the more realistic application of antibody conjugates. We have, therefore, initiated a programme aimed at photochemical targeting of ovarian cancer cells using monoclonal antibody–photosensitiser conjugates administered intraperitoneally. The photosensitiser chlorin $e_6$ monoethylendiamine monamide (CMA) was conjugated site specifically via polyglutamic acid (PGA) to a murine monoclonal antibody, OC125 (Goff *et al.*, 1991). OC125 recognises the antigen CA 125 (Bast *et al.*, 1981), which is expressed on 80% of non-mucinous ovarian cancers (Bast *et al.*, 1981; Davis *et al.*, 1986; Miotti *et al.*, 1987). The conjugate was significantly more phototoxic to ovarian cancer cells both *in vitro* (cell lines) and *ex vivo* (from cancer patients) (Goff *et al.*, 1991, 1992), while exhibiting no phototoxicity to CA 125-negative cells.

The present study is an extension of our previous *in vitro* and *ex vivo* photodynamic experiments and reports the results of our initial *in vivo* investigations. As a first step in this direction, we investigated the biodistribution and tumour uptake of the OC125-IC in a murine ascites ovarian cancer model by photosensitiser extraction and fluorescence spectro-

Correspondence: T. Hasan, Massachusetts General Hospital, Wellman Laboratories of Photomedicine, WEL 224, 55 Fruit Street, Boston, MA 02114, USA.
Received 28 September 1993; and in revised form 7 January 1994.

scopy. Preliminary studies evaluating the effectiveness of PDT using this conjugate were also conducted in the same model.

### Materials and methods

### *Tumour cells*

NIH:OVCAR3 cells were a generous gift from Centocor (Malvern, PA, USA). This cell line was derived from the ascites of a patient with ovarian cancer (Hamilton *et al.*, 1983). Cells were grown in RPMI-1640 medium supplemented with heat-inactivated fetal bovine serum and kept in an incubator at 37°C in an atmosphere of 5% carbon dioxide. For tumour transplantation, cells were trypsinised (trypsin-EDTA, Gibco, Grand Island, NY, USA), centrifuged at 1,000 r.p.m. for 10 min (model 6000B, Sorvall Centrifuges, Dupont, Wilmington, DE, USA) and resuspended in 1 ml of phosphate-buffered saline (PBS) (Gibco) for intraperitoneal injection.

### *Tumour model*

Experiments were carried out in a murine model for ovarian cancer developed by Hamilton *et al.* (1984). Balb/c athymic nude mice (Charles River Breeding Laboratories) were given intraperitoneal injections of $30 \times 10^6$ NIH:OVCAR3 cells. This results in the development of serosal metastases similar to that of ovarian cancer in humans. Disease progression is characterised by the development of massive ascites and extensive intraperitoneal tumours. Within 6 weeks, animals develop clinical evidence of ascites, at which time biodistribution studies are performed.

### *Photosensitisers*

CMA was obtained from Porphyrin Products (Logan, UT, USA) and the PGA was obtained from Sigma (St Louis, MO, USA). The monoclonal antibody OC125 was a gift from Centocor, and was coupled site specifically at its carbohydrate moiety with CMA-bound PGA as previously described to form the OC125-PGA-CMA IC (Goff *et al.*, 1991).

### *Biodistribution*

CMA was injected intraperitoneally at a dose of 2.0 mg per kg body weight; the IC and PGA-CMA were injected at CMA equivalents matched for identical absorption at 656 nm with the free CMA solution. Following injection of the photosensitiser, animals ($n$ = 5–7) were sacrificed by cervical dislocation at 3, 6, 12, 24, 48, 76 and 168 h. The ascites containing tumour cells, kidney, liver, skin, small intestine and plasma were harvested from the animals. For the intestine specimen, the whole wall including mucosa, submucosa, muscle and serosa was sampled. Wet tissue samples were weighed and frozen at −70°C. For extraction of the photosensitiser the samples were homogenised (homogeniser model PT 10/35, Brinkman Instruments, Westbury, NY, USA) in 3 ml of 0.1 M sodium hydroxide and centrifuged at 17,000 r.p.m. (Sorvall RC-5B, refrigerated superspeed centrifuge, Dupont Instruments) for 20 min (Bachor *et al.*, 1992). The fluorescence of the supernatant was measured with a spectrofluorimeter (Fluorolog 2, Spex Industries, Edison, NJ, USA) and compared with that of a standard solution of CMA, PGA-CMA or OC125-PGA-CMA (IC). The excitation wavelength was 400 nm and emission spectra were monitored between 500 and 700 nm. The fluorescence of tissue samples from untreated animals was used for background correction. The quantitative nature of the extraction procedure was established by spectrophotometric readings of successive extracts and by comparison with extracts of known mixes of PSs and tissues.

### *Photodynamic treatment* in vivo

Mice were injected with 30 million NIH:OVCAR3 cells. Seven days after injection, animals were given an intraperitoneal injection of IC or CMA (at 0.5 and 2.0 mg kg$^{-1}$ CMA equivalent as described above) followed by irradiation 24 h later. Controls were injected with sterile PBS. Animals in one group (PS dose 2.0 mg kg$^{-1}$) were immobilised and given external whole-abdomen irradiation (field size approximately 3.5 cm diameter) with an argon ion pumped dye laser (Coherent, Palo Alto, CA, USA) at a wavelength of 656 nm ($\lambda_{max}$ for CMA). External irradiation was used in these initial studies because there was no requirement for sedation or violation of the peritoneal cavity. Also, in nude mice, the abdominal wall is thin (<2 mm) and presents minimal barrier to 656 nm irradiation (Svaasand *et al.*, 1990). The power density measured at the skin surface was 40–70 mW cm$^{-2}$, and the fluence administered was 20 and 30 J cm$^{-2}$. The second group (PS dose 0.5 mg kg$^{-1}$) was sedated with sodium pentabarbital and intraperitoneal irradiation was carried out according to the method of Tochner *et al.* (1985, 1986) at 656 nm, 50 mW and 1 min per quadrant. Animals were treated with PS and light every 48 h for a total of three treatments. A quantitative assessment of the extent of ovarian cancer cell destruction was performed *ex vivo* for both groups of animals. One hour after irradiation the ascites was harvested with a 15 gauge needle. RBCs were lysed with 0.83% amonium chloride (Aldrich Chemical, Milwaukee, WI, USA). Ovarian cancer cells were washed in sterile Dulbecco's PBS (Gibco, Grand Island, NY, USA) and placed in 35 mm Petri dishes at a concentration of 150,000 cells ml$^{-1}$ in RPMI-1640 medium (Gibco) containing 10% fetal bovine serum. Forty-eight hours after plating, the survival of treated and control *ex vivo* cultures was determined by the 3-(4,5-dimethylthiazol-2-yl)-2,5 diphenyl terazolium bromide (MTT) colorimetric assay (Mosmann, 1983).

### Results

### *Photosensitisers*

The qualitative UV-visible absorption spectra for free CMA and the OC125-PGA-CMA (IC) are shown in Figure 1. The spectra are essentially similar, though the IC shows a 3–5 nm blue shift compared with free CMA.

### *Biodistribution*

The tumour content of CMA, PGA-CMA and IC as a function of time are shown in Figure 2. The peak concentra-

**Figure 1** UV-visible absorption spectra of CMA and IC dissolved in PBS. The CMA concentration was 7.1 μM in PBS and the IC concentration was arbitrarily adjusted to display the spectrum clearly.

tion for CMA occurs at 24 h and slowly declines over time. The tissue distribution of free CMA is shown in Table I. For blood, there is a brief peak at 6 h, following which the level drops to almost zero. For liver and kidney there is almost no uptake of CMA. In the skin and intestines there is some uptake initially at 3, 6 and 12 h, but the tissue concentrations drop off by 24 h.

The biodistribution of PGA-CMA is shown in Table II. Peak tumour tissue concentrations of PGA-CMA are found at 3 h and slowly decline over time. As for other tissues, blood, liver and kidney have minimal uptake. However, skin and intestine retain significant amounts of PGA-CMA and have tissue concentrations similar to tumour levels. The tumour to skin and intestine ratios were in general close to 1 at most time points. At 3 and 24 h, tumour to skin ratios of up to 3 were obtained. At these time points, the tumour to intestine ratios were 1.6 and 0.8 respectively.

The tissue distribution of the IC is shown in Table III. The peak tumour concentration (3.18 ± 1.38 nmol per g of tissue) also occurs at 24 h, as with CMA (Figure 2); however, the mean tissue concentration of the IC is twice that of free CMA. As levels decline in the tumour, liver and kidney concentrations increase, with peak tissue concentration of IC occurring at 168 h in these tissues (1.03 ± 0.34 nmol $g^{-1}$ for liver and 0.69 ± 0.35 for kidney). Blood concentrations are low at all time points. The levels in the intestine are modestly elevated and peak at 12 h. At 24 h, the concentration of IC in tumour is 3.5 times greater than in the intestines, but IC remains in the intestines even at 336 h.

**Figure 2** Balb/c nude mice with ovarian ascites tumours were injected intraperitoneally with 2.0 mg $kg^{-1}$ CMA (●), PGA-CMA (□) or IC (▲) (for PGA-CMA and IC, CMA equivalently matched by absorption at 656 nm). The curves represent the tumour content of each compound in nmol $g^{-1}$ tissue over time.

Tumour to non-tumour ratios for free CMA, PGA-CMA and IC are presented in Table IV for all measured time points and in Figure 3 for the 3, 12 and 24 h time points. For the IC, the peak tumour concentration (3.18 ± 1.38 nmol $g^{-1}$ tissue) occurs at 24 h. At that time, tumour to non-tumour ratios are 6.8 for blood, 6.5 for liver, 7.2 for kidney, 5.7 for skin and 3.5 for intestine. At 48 and 72 h tumour to non-tumour ratios decrease for all tissues except blood; however, the ratios are all still above 2. For CMA, tissue concentrations are generally about half those for IC; peak tumour concentrations (1.67 ± 0.40 nmol $g^{-1}$ tissue) occur at 24 h, as with the IC. Tumour to non-tumour ratios appear to be high. However, the lower absolute concentrations border the detection limits.

*Photodynamic therapy*

Once the biodistribution studies had identified 24 h following injection of CMA or IC as an appropriate starting time for irradiation, *in vivo–in vitro* experiments were carried out to assess the extent of photodynamic destruction of the ascites tumour cell population. Ascites harvested from tumour-bearing animals after treatment with IC or CMA and light was assayed for survival by the MTT assay and compared with untreated controls. The results are presented in Table V. Animals were initially treated with a single external light exposure. At a dose of 2 mg $kg^{-1}$ and 20 J $cm^{-2}$ 40% of the cells treated with the IC and 70% of cells treated with CMA survived. At 30 J $cm^{-2}$, cell survival was 14% and 56% for IC and CMA treatment respectively. However, even at the lower light dose, 30–50% of the animals treated with IC or with CMA died secondary to treatment toxicity. Histological profiles of dead animals 24 h post PDT showed vascular congestion in liver, kidney, lung and spleen similar to those

**Table I** Mean concentrations (nmol $g^{-1}$ tissue) of free CMA in mice[a] (± s.d.)

| Time (h) | Ascites | Blood | Liver | Kidney | Skin | Intestine |
|---|---|---|---|---|---|---|
| 3 | 0.91 ± 0.37 | 0.18 ± 0.16 | 0.07 ± 0.04 | 0.04 ± 0.03 | 0.31 ± 0.27 | 0.54 ± 0.78 |
| 6 | 1.39 ± 0.73 | 0.43 ± 0.05 | 0.08 ± 0.05 | 0.06 ± 0.04 | 0.32 ± 0.19 | 0.37 ± 0.17 |
| 12 | 1.28 ± 0.79 | 0.01 ± 0.007 | 0.07 ± 0.05 | 0.03 ± 0.03 | 0.21 ± 0.13 | 0.12 ± 0.12 |
| 24 | 1.67 ± 0.40 | 0.02 ± 0.02 | 0.06 ± 0.04 | 0.03 ± 0.03 | 0.09 ± 0.05 | 0.11 ± 0.12 |
| 48 | 1.50 ± 0.86 | 0.01 ± 0.01 | 0.05 ± 0.03 | 0.02 ± 0.02 | 0.06 ± 0.05 | 0.07 ± 0.08 |
| 72 | 1.12 ± 0.34 | 0.005 ± 0.005 | 0.03 ± 0.02 | 0.02 ± 0.02 | 0.06 ± 0.04 | 0 |

[a]Balb/c nude mice ($n$ = 5–7) with ovarian ascites tumours were injected intraperitoneally with 2 mg $kg^{-1}$ CMA. Animals were sacrificed at various times. The amount of CMA was quantitated by extraction followed by fluorescence spectroscopy.

**Table II** Mean concentrations (nmol $g^{-1}$ tissue) of PGA-CMA in mice[a] (± s.d.)

| Time (h) | Ascites | Blood | Liver | Kidney | Skin | Intestine |
|---|---|---|---|---|---|---|
| 3 | 3.91 ± 2.51 | 0.55 ± 0.58 | 0.55 ± 0.03 | 0.30 ± 0.46 | 1.45 ± 0.37 | 2.46 ± 0.38 |
| 6 | 2.44 ± 0.33 | 0.67 ± 0.3 | 0.65 ± 0.46 | 0.23 ± 0.32 | 2.88 ± 1.45 | 2.44 ± 2.45 |
| 12 | 2.42 ± 1.42 | 0.14 ± 0.10 | 1.08 ± 0.63 | 0.47 ± 0.41 | 3.32 ± 1.02 | 1.14 ± 0.44 |
| 24 | 2.18 ± 0.39 | 0.28 ± 0.05 | 0.46 ± 0.13 | 0.24 ± 0.26 | 1.30 ± 0.78 | 2.75 ± 0.24 |
| 48 | 1.22 ± 0.29 | 0.20 ± 0.12 | 0.60 ± 0.15 | 0.14 ± 0.25 | 1.25 ± 0.11 | 0.91 ± 0.53 |
| 72 | 1.34 ± 0.05 | 0.46 ± 0.044 | 0.42 ± 0.10 | 0.13 ± 0.12 | 1.87 ± 0.42 | 1.93 ± 1.06 |
| 168 | 0.51 ± 0.10 | 0.16 ± 0.09 | 0.32 ± 0.10 | 0.09 ± 0.16 | 1.10 ± 1.07 | 2.78 ± 1.56 |

[a]Balb/c nude mice ($n$ = 5–7) with ovarian ascites tumours were injected intraperitoneally with 2 mg $kg^{-1}$ PGA-CMA (CMA equivalent determined by matching 656 nm aborbance to a standard CMA solution). Animals were sacrificed at various times. The amount of PGA-CMA was quantitated by extraction followed by fluorescence spectroscopy.

**Table III** Mean concentrations (nmol $g^{-1}$ tissue) of IC in mice[a] (± s.d.)

| Time (h) | Ascites | Blood | Liver | Kidney | Skin | Intestine |
|---|---|---|---|---|---|---|
| 3 | 2.84 ± 2.07 | 0.36 ± 0.25 | 0.43 ± 0.29 | 0.35 ± 0.25 | 0.31 ± 0.30 | 1.34 ± 1.03 |
| 6 | 2.58 ± 1.99 | 0.34 ± 0.26 | 0.47 ± 0.34 | 0.27 ± 0.14 | 0.69 ± 0.39 | 0.86 ± 0.60 |
| 12 | 2.36 ± 1.16 | 0.40 ± 0.36 | 0.47 ± 0.26 | 0.57 ± 0.28 | 0.59 ± 0.26 | 2.14 ± 1.04 |
| 24 | 3.18 ± 1.38 | 0.47 ± 0.19 | 0.49 ± 0.16 | 0.44 ± 0.30 | 0.56 ± 0.23 | 0.92 ± 0.44 |
| 48 | 2.25 ± 1.10 | 0.19 ± 0.11 | 0.74 ± 0.48 | 0.50 ± 0.28 | 1.03 ± 0.27 | 0.91 ± 0.62 |
| 72 | 2.21 ± 0.55 | 0.14 ± 0.09 | 0.46 ± 0.32 | 0.48 ± 0.30 | 0.58 ± 0.37 | 1.11 ± 0.95 |
| 168 | 0.80 ± 0.45 | 0.04 ± 0.05 | 1.03 ± 0.34 | 0.69 ± 0.35 | 0.95 ± 0.17 | 1.30 ± 0.45 |
| 336 | 0.32 | 0.13 ± 0.01 | 0.70 ± 0.19 | 0.32 ± 0.04 | 0.31 ± 0.18 | 0.91 ± 0.70 |

[a]Balb/c nude mice ($n = 5-7$) with ovarian ascites tumours were injected intraperitoneally with 2 mg $kg^{-1}$ IC (OC125-PGA-CMA at CMA equivalent determined by aborbance matching at 656 nm). Animals were sacrificed at various times. The amount of IC was quantitated by extraction followed by fluorescence spectroscopy.

**Figure 3** Biodistribution of PGA-CMA (■, skin; □, intestine) and IC (▨, skin; ▧, intestine) 3, 12 and 24 h after intraperitoneal administration. Data are presented as tumour to skin and tumour to intestine ratios.

**Table IV** Tumour to non-tumour ratios[a]

| Time (h) | Blood | Liver | Kidney | Skin | Intestine |
|---|---|---|---|---|---|
| *CMA* | | | | | |
| 3 | 5.0 | 13.0 | 22.7 | 2.9 | 1.7 |
| 6 | 3.2 | 17.4 | 23.2 | 4.3 | 3.8 |
| 12 | 128.0 | 18.3 | 42.7 | 6.1 | 10.7 |
| 24 | 83.5 | 27.8 | 55.7 | 18.5 | 15.2 |
| 48 | 150.0 | 30.0 | 75.0 | 25.0 | 21.4 |
| 72 | 224.0 | 37.3 | 56.0 | 18.7 | – |
| *PGA-CMA* | | | | | |
| 3 | 7.1 | 7.1 | 11.1 | 2.7 | 1.6 |
| 6 | 3.6 | 3.7 | 10.6 | 0.8 | 1.0 |
| 12 | 17.3 | 2.2 | 5.1 | 0.7 | 2.1 |
| 24 | 7.8 | 4.7 | 9.0 | 3.0 | 0.8 |
| 48 | 6.1 | 2.0 | 8.7 | 1.0 | 1.3 |
| 72 | 5.7 | 5.8 | 18.7 | 1.3 | 1.3 |
| 168 | 3.1 | 1.6 | 5.6 | 0.5 | 0.2 |
| *OC125-PGA-CMA* | | | | | |
| 3 | 7.9 | 6.6 | 8.1 | 9.2 | 2.1 |
| 6 | 7.6 | 5.5 | 9.5 | 3.7 | 3.0 |
| 12 | 5.9 | 5.0 | 4.1 | 4.0 | 1.1 |
| 24 | 6.8 | 6.5 | 7.2 | 5.7 | 3.5 |
| 48 | 11.8 | 3.0 | 4.5 | 2.2 | 2.5 |
| 72 | 15.8 | 4.8 | 4.6 | 3.8 | 2.0 |
| 168 | 20.0 | 0.8 | 1.2 | 0.8 | 0.7 |
| 336 | 2.5 | 0.4 | 1.0 | 1.0 | 0.4 |

[a]Numbers represent the ratio of CMA, PGA-CMA, or OC125-PGA-CMA (IC) in ascites tumour cells compared with normal tissue.

described by Ferrario and Gomer (1990). Because of the toxicity experienced by these animals, the IC and CMA dose was lowered to 0.5 mg $kg^{-1}$ and intraperitoneal irradiations were carried out at doses of approximately 5 J $cm^{-2}$ per treatment for a total of 15 J $cm^{-2}$. Following three such PDT treatments (of both PS and light) the fraction of viable tumour cells in the ascites of the animals treated with IC was approximately 5% of control. For those treated with free CMA the viable tumour cells were 15% of control. Under these conditions, no toxicity to animals was noted.

**Table V** *In vivo–in vitro* experiments with ascites-bearing mice[a] (± s.d.)

| | IC | CMA |
|---|---|---|
| 2 mg $kg^{-1}$–20 J $cm^{-2}$ | 40.3 ± 14.9 | 71.7 ± 4.8 |
| 2 mg $kg^{-1}$–30 J $cm^{-2}$ | 14.1 ± 10.1 | 56.5 ± 24.3 |
| 0.5 mg $kg^{-1}$∼5 J $cm^{-2}$ i.p. | 5 ± 1.5 | 15 ± 5.9 |

[a]Numbers represent percentage of cells which survived as determined by MTT assay as compared with untreated controls. Animals administered 2 mg $kg^{-1}$ PS were irradiated externally. Animals treated with 0.5 mg $kg^{-1}$ PS were irradiated intraperitoneally. In each group there were 7–10 animals.

### Discussion

Monoclonal antibody immunoconjugates are being investigated for use in tumour localisation and treatment in a variety of malignant diseases. Indium-111-labelled OC125 has been used for tumour localisation in ovarian cancer patients (Chatal *et al.*, 1989). Doxorubicin linked to OC125 has shown improved cytotoxicity *in vitro* against an ovarian cancer cell line compared with unconjugated doxorubicin (Sweet *et al.*, 1989). Because ovarian cancer is usually limited to the peritoneal cavity, intraperitoneal injections of ICs may be the preferred route of administration, as demonstrated by several previous studies (Ward & Wallace, 1987; Ward *et al.*, 1987; Ward & Piko, 1987). Indeed, with radioimmunoconjugates intraperitoneal injection results in higher tumour uptake, presumably because of the greater ability of IC to access its tumour antigenic target (Ward *et al.*, 1987; Finkler *et al.*, 1989; Thedrez *et al.*, 1989) prior to RES capture. With *intravenous* injection of $^{111}$In-OC125 in an ascitic murine model similar to the one used in this study, uptake in tumour was significantly lower than after administration by the intraperitoneal route, while the RES uptake was high. Tumour to non-tumour ratios were also reported to be significantly lower after intravenous injection and the amount of the radioisotopic antibody in normal tissue, in particular the organs of the RES, was high, with consequent toxicity (Thedrez *et al.*, 1989). Based on such rationale, radioimmunotherapy using intraperitoneally injected $^{111}$I-labelled OC125 has been studied in phase I trials to treat recurrent ovarian carcinoma (Finkler *et al.*, 1989; Muto *et al.*, 1992).

Toxicity was minimal, but tumour progression was noted in the majority of patients and no complete responses were obtained. For ligand doses which are curative or inhibit tumour progression, systemic toxicity from bound toxins or radioisotopes remains a significant problem because of the lack of specificity of most monoclonal antibodies. Clearly, other modes of treatments are required for effective and less toxic cure or palliation of ovarian cancer.

Using monoclonal antibodies to deliver photosensitisers selectively is a relatively recent treatment approach that has a potential for minimising normal tissue toxicity from antibody-bound toxic ligands. Since the first such study by Mew *et al.* (1983), the approach has been further developed (Hasan *et al.*, 1989*a–c*, 1992, 1993; Jiang *et al.*, 1992, 1993). However, reports of *in vivo* examination of this approach have been limited (Mew *et al.*, 1983; Hasan *et al.*, 1989*a*; Jiang *et al.*, 1993). In the present study we characterise the biodistribution and toxicity of this IC in a murine ovarian cancer model that has many similarities to the clinical course of ovarian cancer in humans.

The tumour to non-tumour ratio may determine the optimal time for treatment. Ideally, there should be high photosensitiser concentration in tumours with low concentrations in surrounding normal tissues. Although the photosensitiser concentrations for the PGA-CMA and the IC were similar, the tumour to non-tumour ratios for skin and intestine for PGA-CMA are close to unity at several time points (Figure 3). At points when tumour–skin ratios of close to 3 were obtained for PGA-CMA, the tumour–intestine ratios were low (1.6 and 0.8 at 3 and 24 h respectively). Since the phase I trials with HPD suggested bowel perforation as a major complication, this compound seemed unattractive for treatment because of increased risk of toxicity to these organs. For the IC the highest tumour to non-tumour ratios occurred at 24–48 h. These ratios are similar to what other investigators have found for radiolabelled OC125 (Thedrez *et al.*, 1989). Apparently linking CMA to OC125 does not significantly affect the binding of the monoclonal antibody *in vivo*. The unexpected result from this study was the apparent very high tumour to non-tumour ratios seen with free CMA at 24, 48 and 72 h.

Despite the apparent high tumour to non-tumour ratios for free CMA (~10-fold), CMA was significantly less phototoxic to ovarian cancer cells *in vivo*. Tumour cell survival after *in vivo* treatment with identical doses of IC or CMA and light indicates that the percentage of tumour cells surviving after IC treatment is 2- to 4-fold less than with the free CMA (Table V). This reduced cytotoxicity of CMA to tumour cells could be caused by the smaller absolute CMA concentrations in tumour cells or possibly by the presence of a non-photoactive metabolite of CMA with similar spectral properties. For another chlorin derivative, mono-L-aspartyl-chlorin $e_6$, Gomer and Ferrario (1990) found that the tumour concentration and tumour to non-tumour ratios were also not predictive of optimal treatment times and toxicity. In their murine model of subcutaneous mammary cancer, the most significant tumour necrosis occurred when plasma levels of the chlorin derivative were high, suggesting vascular-mediated damage as the primary target. Since ovarian cancer is a disease that spreads superficially along peritoneal surfaces and has minimal vascularity, particularly in the model used in this study, a primary vascular response is probably not relevant and cannot be responsible for the lower CMA-induced phototoxicity.

The apparent high preferential localisation of CMA in tumours would not have been anticipated from *in vitro* and *ex vivo* experiments in which CMA showed no selective cytotoxicity (Goff *et al.*, 1991, 1992). The reasons for this observation are not obvious. The tumour cells being analysed in this study are ascitic and are either single-cell suspensions or clumps of cells. As such, the neovasculature and the lymphatic systems are probably not well developed and are therefore unlikely to be responsible for the preferential tumour retention. It is possible that preassociation with physiological macromolecular components *in vivo* causes this differential (Kongshaug *et al.*, 1989). Alternatively, the extracted species that is being interpreted as CMA from fluorescence data is a metabolite with similar optical properties.

Comparison of *in vivo* and *in vitro* photodestruction reveals that *in vivo* PDT is inefficient compared with *in vitro*, probably because light delivery is more effective *in vitro*. For similar intracellular photosensitiser content (~2 fmol per cell) and incident fluence, virtually a 100% cytotoxicity was observed for *in vitro* irradiations (Goff *et al.*, 1991). These data also suggest a dominant cellular mechanism since no increased phototoxicity is observed *in vivo*, as is the case for sensitisers with a strong vascular component (Henderson & Dougherty, 1992).

In summary, our experiments show that OC125 photoimmunoconjugates administered intraperitoneally may be useful in the palliative PDT of ovarian carcinoma. Because ovarian cancer is predominantly a disease with serosal spread limited to the peritoneal cavity, it is ideally suited for photochemotherapy. The peritoneal surface can be exposed to laser irradiation both intraoperatively and during laparoscopy. Small residual disease following cytoreductive surgery, microscopic disease or diaphragmatic studding could readily be treated by this modality.

Our study suggests that for optimal tumoricidal effect, PDT may need to be administered in multiple treatments. This is similar to the results of Tochner *et al.* (1985, 1986), who used haematoporphyrin-based PDT to treat a murine ovarian cancer model. With a single treatment, even at high sensitiser and light doses, approximately 50% of the tumour cells survived. Multiple treatments administered intraperitoneally were significantly more effective at lower doses. More quantitative light delivery and dosimetry are also necessary to reduce phototoxic death seen in this study from the generalised external irradiation of the peritoneal cavity. There is also ample room for the improvement of tumour uptake of antibody-bound photosensitiser. The modest differential observed between tumour and non-tumour tissues with the IC in this study is probably due to the inefficiency of OC125. This antibody binds to some normal tissues (Niloff *et al.*, 1984; Miotti *et al.*, 1987; Talbot *et al.*, 1989) and the antigen it binds to is also shed efficiently. The use of more specific antibodies or antibody cocktails and/or fragments should improve the efficacy of PDT. Chimeric antibodies may further enhance uptake and reduce the human anti-mouse antibody response clinically (Muto *et al.*, 1990). Aspects of the above issues are currently under investigation in our laboratory.

We thank Dr K. Schomacker for advice regarding the light delivery and dosimetry aspects of this study and Coherent, Inc. (Palo Alto, CA, USA) for loan of the lasers. This work was supported in part by the American College of Obstetricians and Gynecologists, the American Cancer Society, Aid for Cancer Research, the Vincent Memorial Hospital and National Institutes for Health Grant No. 1R01 AR40352-01A1.

### References

BACHOR, R., FLOTTE, T., SCHOLZ, M., DRETLER, S. & HASAN, T. (1992). Comparison of intravenous and intravesical administration of chloro-aluminum sulfonated phthalocyanine for photodynamic treatment in a rat bladder cancer model. *J. Urol.*, **147**, 1404–1410.

BAST Jr, R.C., FEENEY, M., LAZARUS, H., NADLER, L.M., COLVIN, R.B. & KNAPP, R.C. (1981). Reactivity of a monoclonal antibody with human ovarian carcinoma. *J. Clin. Invest.*, **68**, 1331–1337.

BAST Jr. R.C., KLUG, T.L., ST. JOHN, E., JENISON, E., NILOFF, J.M., LAZARUS, H., BERKOWITZ, R.S., LEAVITT, T., GRIFFITHS, C.T., PARKER, L., ZURAWSKI Jr, V.R. & KNAPP, R.C. (1982). A radioimmunoassay using a monoclonal antibody to monitor the course of epithelial ovarian cancer. *N. Engl. J. Med.*, **309**, 883-887.

BORING, C.C., SQUIRES, T.S. & TONG, T. (1993). Cancer statistics. CA-A. *Cancer J. Clin.*, **43**, 7-26.

CHATAL, J.F., SACCAVINI, J.C., GESTIN, J.F., THEDREZ, P., CURTET, C., KREMER, M., GUERREAU, D., NOLIBE, D., FUMOLEAU, P. & GUILLAR, Y. (1989). Biodistribution of indium-III-labeled OC125 monoclonal antibody intraperitoneally injected into patients operated on for ovarian carcinomas. *Cancer Res.*, **49**, 3087-3094.

DAVIS, H.M., ZURAWSKI Jr, V.R., BAST Jr, R.C. & KLUG, T.L. (1986). Characterization of the OC125 antigen associated with human epithelial ovarian carcinomas. *Cancer Res.*, **46**, 6143-6148.

DELANEY, T.F., SINDELAR, W.F., TOCHNER, Z., SMITH, P.D., FRIAUF, W.S., THOMAS, G., DACHOWSKI, L., COLE, J.W., STEINBERG, S.M. & GLATSTEIN, E. (1993). Phase I study of debulking surgery and photodynamic therapy for disseminated intraperitoneal tumors. *Int. J. Radiat. Oncol. Biol. Phys.*, **25**, 445-457.

DOUGHERTY, T.J. (1987). Photosensitizers: therapy and detection of malignant tumors. *Photochem. Photobiol.*, **45**, 879-889.

FERRARIO, A. & GOMER, C.J. (1990). Systemic toxicity in mice induced by localized porphyrin photodynamic therapy. *Cancer Res.*, **50**, 539-543.

FINKLER, N.J., MUTO, M.G., KASSIS, A.I., WEADOCK, K., TUMEH, S.S., ZURAWSKI, V.R. & KNAPP, R.C. (1989). Intraperitoneal radiolabeled OC125 in patients with advanced ovarian cancer. *Gynecol. Oncol.*, **34**, 339-344.

GOFF, B.A., BAMBERG, M. & HASAN, T. (1991). Photoimmunotherapy of human ovarian carcinoma cells *ex vivo*. *Cancer Res.*, **51**, 4762-4767.

GOFF, B.A., BAMBERG, M. & HASAN, T. (1992). Experimental photodynamic treatment of ovarian carcinoma cells with immunoconjugates. *Antibody Immunoconj. Radiopharm.*, **5**, 191-199.

GOMER, C. (1991). Preclinical examination of first and second generation photosensitizers used in photodynamic therapy. *Photochem. Photobiol.*, **54**, 1093-1107.

GOMER, C.J. & FERRARIO, A. (1990). Tissue distribution and photosensitizing properties of mono-L-aspartyl chlorin $e_6$ in a mouse tumor model. *Cancer Res.*, **50**, 3985-3990.

HAMILTON, T.C., YOUNG, R.C., MCKOY, W.M., GROTZINGER, K.R., GREEN, J.A., CHU, E.W., WHANG-PENG, J., ROGAN, A.M., GREEN, W.R. & OZOLS, R.F. (1983). Characterization of a human ovarian carcinoma cell line (NIH:OVCAR3) with androgen and estrogen receptors. *Cancer Res.*, **43**, 5379-5389.

HAMILTON, T.C., YOUNG, R.C., LOUIS, K.G., BEHRENS, B.C., MCKOY, W.M., GROTZINGER, K.R. & OZOLS, R.F. (1984). Characterization of a xenograft model of human ovarian carcinoma which produces ascites and intraabdominal carcinomatosis in mice. *Cancer Res.*, **44**, 5286-5290.

HASAN, T. (1992). Photosensitizer delivery mediated by macromolecular carrier systems. In *Photodynamic Therapy: Basic Principles and Clinical Applications*, Henderson, B. & Dougherty, T. (eds) pp. 187-200. Marcel Dekker: New York.

HASAN, T. (1993). Photochemical targeting of cancer with macromolecular delivery systems. In *Cancer Therapy into the Twenty-First Century* Huber, B. (ed.). Springer: New York, in press.

HASAN, T., CHEN, N., ANDERSON, T., DEAK, M.R., LINDEN, K., GRANSTEIN, R., ZURAWSKI, V.R. & FLOTTE, T. (1989*a*). Immunologic targeting of cancer cells. *Fundamentals of Photodynamic Therapy, SPIE Proceedings*, **1065**, 80-86.

HASAN, T., LIN, A., YARMUSH, D., OSEROFF, A. & YARMUSH, M. (1989*b*). Monoclonal antibody-chromophore conjugates as selective phototoxins. *J. Controlled Release*, **10**, 107-117.

HASAN, T., LIN, C.W. & LIN, A. (1989*c*). Laser-induced selective cytotoxicity using monoclonal antibody-chromophore conjugates. *Prog. Clin. Biol. Res.*, **288**, 471-477.

HENDERSON, B.W. & DOUGHERTY, T.J. (1992). How does photodynamic therapy work? *Photochem. Photobiol.*, **55**, 145-157.

JIANG, F.N., JIANG, S., LIU, D., RICHTER, A. & LEVY, J.G. (1990). Development of technology for linking photosensitizers to a model monoclonal antibody. *J. Immunol. Methods*, **134**, 139-149.

JIANG, F.N., ALLISON, B., LIU, D. & LEVY, J.G. (1992). Enhanced photodynamic killing of target cells by either monoclonal antibody or low density lipoprotein mediated delivery systems. *J. Controlled Release*, **19**, 41-58.

JIANG, F.N., RICHTER, A.M., JAIN, A.K., LEVY, J.G. & SMITS, C. (1993). Biodistribution of a benzoporphyrin derivative-monoclonal antibody conjugate in A549 tumor bearing nude mice. *Biotechnol. Ther.*, **4**, 43-61.

KONGSHAUG, M., MOAN, J. & BOWN, S.B. (1989). The distribution of porphyrins with different tumour localising ability among human plasma proteins. *Br. J. Cancer*, **59**, 184-188.

MARCUS, S.L. (1992). Photodymaic therapy of human cancer. *Proc. IEEE*, **80**, 869-889.

MEW, D., WAT, C.K., TOWERS, G.H. & LEVY, J.G. (1983). Photoimmunotherapy: treatment of animal tumors with tumor-specific monoclonal antibody-hematoporphyrin conjugates. *J. Immunol.*, **130**, 1473-1477.

MEW, D., LUM, V., WATT, C.K., TOWERS, G.H., SUN, C.H., WALTER, R.J., WRIGHT, W., BERNS, M.W. & LEVY, J.G. (1985). Ability of specific monoclonal antibodies and conventional antisera conjugated to hematoporphyrin to label and kill selected cell lines subsequent to light activation. *Cancer Res.*, **45**, 4380-4386.

MIOTTI, S., CANEVARI, S., MENARD, S., MEZZANZANICA, D., PORRO, G., PUPA, S.M., REGAZZONI, M., TAGLIABUE, E. & COLNAGHI, M.I. (1987). Characterization of human ovarian carcinoma-associated antigens defined by novel monoclonal antibodies with tumor-restricted specificity. *Int. J. Cancer*, **39**, 297-303.

MOSMANN, T. (1983). Rapid colorimetric assay for cellular growth and survival: application to proliferation and cytotoxic assays. *J. Immunol. Methods*, **65**, 55-63.

MUTO, M.G., FINKLER, N.J., KASSIS, A.I., LEPISTO, E.M. & KNAPP, R.C. (1990). Human anti-murine antibody responses in ovarian cancer patients undergoing radioimmunotherapy with the murine monoclonal antibody OC125. *Gynecol. Oncol.*, **38**, 244-248.

MUTO, M.G., FINKLER, N.J., KASSIS, A.I., HOWES, A.E., ANDERSON, L.L., LAU, C.C., ZURAWSKI, V.R., WEADOCK, K., TUMEH, S.S., LAVIN, P. & KNAPP, R.C. (1992). Intraperitoneal radioimmunotherapy of refractory ovarian carcinoma utilizing iodine-131-labeled monoclonal antibody OC125. *Gynecol. Oncol.*, **45**, 265-272.

NILOFF, J.M., KNAPP, R.C., SCHAETEL, E., REYNOLDS, C. & BAST Jr, R.C. (1984). CA125 level in obstetric and gynecologic patients. *Obstet. Gynecol.*, **64**, 703-707.

OSEROFF, A., OHUOHA, D., HASAN, T., BOMMER, J.C. & YARMUSH, M.L. (1986). Antibody-targeted photolysis: selective photodestruction of human T cell leukemia cells using monoclonal antibody-chlorin $e_6$ conjugates. *Proc. Natl Acad. Sci. USA*, **83**, 8744-8748.

RAKESTRAW, S.L., TOMPKINS, R.G. & YARMUSH, M.L. (1990). Antibody-targeted photolysis: *in vitro* studies with SN(iv) chlorin $e_6$ covelently bound to monoclonal antibodies using a modified dextran carrier. *Proc. Natl Acad. Sci. USA*, **87**, 4217-4221.

SVAASAND, L.O., MORRIELLI, E., GOMER, C.J. & PROFIO, E. (1990). Optical characteristics of intra-ocular tumors in the visible and near infra-red. *Proc. SPIE*, **1203**, 2-11.

SWEET, F., ROSKIN, L.O., SOMMERS, G.M. & COLLINS, J.L. (1989). Daunorubicin conjugated to a monoclonal anti-CA125 antibody selectively kills ovarian cancer cells. *Gynecol. Oncol.*, **34**, 305-311.

TALBOT, R.W., JACOBSEN, D.J., NAGORNEY, D.M., MALKASIAN, G.D. & RITTS Jr, R.E. (1989). Temporary elevation of CA125 after abdominal surgical treatment for benign disease and cancer. *Surg. Gynecol. Obstet.*, **168**, 407-412.

THEDREZ, P., SACCAVINI, J.C., NOLIBE, D., SIMOEN, J.P., GUERREAU, D., GESTIN, J.F., KREMER, M. & CHATAL, J.F. (1989). Biodistribution of indium-III-labeled OC125 monoclonal antibody after intraperitoneal injection in nude mice intraperitoneally grafted with ovarian carcinoma. *Cancer Res.*, **49**, 3081-3086.

TOCHNER, Z., MITCHELL, J.B., HARRINGTON, F.S., SMITH, P., RUSSO, D.T. & RUSSO, A. (1985). Treatment of murine intraperitoneal ovarian ascitic tumor with hematoporphyrin derivative and laser light. *Cancer Res.*, **45**, 2983-2987.

TOCHNER, Z., MICHELL, J.B., SMITH, P., GLATSTEIN, E., RUSSO, D.T. & RUSSO, A. (1986). Photodynamic therapy of ascites tumor within the peritoneal cavity. *Br. J. Cancer*, **53**, 733-736.

WAHL, R. & PIKO, C. (1987). Intraperitoneal delivery of radiolabeled monoclonal antibody to IP-induced xenografts of ovarian cancer. *Proc. Am. Assoc. Cancer Res.*, **47**, 4714-4718.

WARD, B.G. & WALLACE, K. (1987). Localization of the monoclonal antibody HMFG2 after intravenous and intraperitoneal injection into nude mice bearing subcutaneous and intraperitoneal human ovarian cancer xenografts. *Cancer Res.*, **47**, 4714–4718.

WARD, B.G., MATHER, S.J., HAWKINS, L.R., CROWTHER, M.E., SHEPARD, J.H., GRANOWSKA, M., BRITTON, K.E. & SLEVIN, M.L. (1987). Localization of radioiodine conjugated to the monoclonal antibody HMFG2 in human ovarian carcinoma: assessment of intravenous and intraperitoneal routes of administration. *Cancer Res.*, **47**, 4719–4723.

WEISHAUPT, K.R., GOMER, C.J. & DOUGHERTY, T.J. (1976). Identification of singlet oxygen as the cytotoxic agent in photoinactivation of a murine tumor. *Cancer Res.*, **36**, 2326–2329.